# HIV self-testing knowledge, attitudes, and practices among Asian-born gay, bisexual, and other men who have sex with men in Australia: a qualitative study

**DOI:** 10.3389/fpubh.2024.1325081

**Published:** 2024-05-02

**Authors:** Ying Zhang, Eric P. F. Chow, Budiadi Sudarto, David Wang, Mark Stoove, Nicholas Medland, Darryl O'Donnell, Phillip Keen, Jason J. Ong, Tiffany R. Phillips

**Affiliations:** ^1^School of Translational Medicine, Faculty of Medicine, Nursing and Health Sciences, Monash University, Melbourne, VIC, Australia; ^2^Melbourne Sexual Health Centre, Alfred Health, Melbourne, VIC, Australia; ^3^Centre for Epidemiology and Biostatistics, Melbourne School of Population and Global Health, The University of Melbourne, Melbourne, VIC, Australia; ^4^Better Health Network, Prahran, VIC, Australia; ^5^Burnet Institute, Melbourne, VIC, Australia; ^6^School of Public Health and Preventive Medicine, Monash University, Melbourne, VIC, Australia; ^7^Australian Research Centre in Sex, Health and Society, La Trobe University, Melbourne, VIC, Australia; ^8^Kirby Institute, University of New South Wales, Sydney, NSW, Australia; ^9^Health Equity Matters, Sydney, NSW, Australia

**Keywords:** HIV, self-test, diagnosis, men who have sex with men, qualitative

## Abstract

**Background:**

Achieving virtual elimination of HIV transmission in Australia requires a combination of high treatment rates and high testing coverage among individuals at risk of acquiring HIV. HIV self-testing (HIVST) is an additional testing approach for key populations.

**Objective:**

We aimed to examine the knowledge, attitudes, and practices of HIVST among Asian-born gay, bisexual and other men who have sex with men (GBMSM).

**Methods:**

This qualitative study used semi-structured interviews of overseas-born GBMSM of Asian background in Australia. Participants were recruited from personal networks, social media platforms, snowballing, and the Melbourne Sexual Health Centre. Twenty-five participants were purposively sampled with a range of ages and previous levels of experience with HIVST. Interview transcripts were imported into Nvivo 12 for data management.

**Results:**

The age of the participants ranged from 19 to 44 years, with a median of 30 years. Most were unaware of HIVST before the interview, and only a few had ever used one. All had limited sexual health knowledge (i.e., HIV testing, PrEP) before they arrived in Australia. Upon learning about HIVST during the interview, many expressed willingness to use HIVST, but in limited circumstances, such as traveling overseas, interim testing while taking on-demand PrEP, and point-of-sex testing. Almost all were open to distributing HIVST to their casual partners or friends, especially those they knew who engaged in high-risk sexual practice (i.e., condomless anal sex) and were not engaged in sexual healthcare. About half still preferred conventional serology testing because of regular HIV testing as part of PrEP prescription and the need for testing for other sexually transmitted infections.

**Conclusion:**

HIVST may be an acceptable additional testing approach for HIV testing among Asian-born GBMSM. Peer education and secondary distribution may help raise HIVST awareness and use.

## 1 Introduction

The goals of the Joint United Nations Program on HIV/AIDS (UNAIDS) include ensuring 95% of people living with HIV (PLHIV) are diagnosed, 95% of those diagnosed are receiving antiretroviral therapy (ART), and viral suppression is achieved by 95% of those receiving ART by 2030 (1). Expanding HIV testing is a crucial entry point toward achieving global HIV status awareness and the elimination HIV transmission. While facility-based HIV testing using conventional serology is recommended, barriers to facility-based HIV testing, such as fear of disclosure, fear of stigma and discrimination, persist (2). In October 2021, the Therapeutic Goods Australia (TGA) approved the Atomo (Atomo diagnostics, Australia) HIV self-test kit, a blood-based HIV self-test, for sale over the counter in pharmacies across Australia to expand HIV testing (3).

The most recent Australian HIV surveillance report indicates a notable reduction in notifications of HIV among Australian-born GBMSM, with a decline of 61% (from 522 in 2012 to 200 in 2021) since 2012. In contrast, the decline among overseas-born GBMSM was comparatively modest, at 31% (from 257 in 2012 to 176 in 2021) (4). Furthermore, Asian-born GBMSM constituted the highest percentage (57%) of late HIV diagnoses among overseas-born individuals in 2021 (4). The observed variation cannot be entirely attributed to sexual practices; rather, they suggest the presence of a potential inherent disparity in the utilization of HIV testing and care among Asian-born GBMSM.

Asian-born GBMSM often face social and cultural stigma surrounding same-sex attraction and HIV in their country of origin (5). Despite experiencing greater acceptance and sexual freedom in Australia, the stigma and shame around their sexual identities have persisted and affected their lived experience in Australia (6). Asian-born GBMSM who have migrated to or hold temporary visas in Australia, such as students or permanent migrants, are subject to marginalization due to their sexual orientation, race, ethnicity and migration status (6, 7). In addition, limited knowledge of navigating the Australian health system, language barriers, and financial constraints may also result in a lack of knowledge about HIV prevention strategies, including testing (7). The internalized stigma and prejudice stemming from their intersectional identities can impact their HIV-related health behaviors (8).

This study aimed to explore the knowledge, attitudes and practices of HIVST among Asian-born GBMSM in Australia and, in turn, to understand the social, medical and cultural issues that may hinder HIVST access. The objectives were to (1) identify the barriers that may prevent Asian-born GBMSM from accessing HIVST, (2) explore strategies that can increase HIVST knowledge and uptake among Asian-born GBMSM, and (3) contrast the experiences of Asian-born GBMSM who have used HIVST with those who have not and identify the social and personal factors that may favor HIVST usage. Findings from this study can be used to tailor interventions aimed at expanding Asian-born GBMSM’s access and use of HIVST in Australia.

## 2 Materials and methods

In this study, a qualitative descriptive methodology was adopted. The qualitative descriptive approach is characterized by its pragmatic nature, as it prioritizes the provision of a description of participants’ experiences and perspectives over an interpretive analysis driven by theory (9, 10). This methodology is frequently used in the healthcare sector to address questions of clinical interest. This approach is useful when there is limited knowledge available on a specific subject (9, 10), and is therefore appropriate for exploring the attitudes and practices of Asian-born GBMSM toward HIVST in Australia. This study has been reported in accordance with the Relevance, Appropriateness, Transparency, and Soundness (RATS) guidelines for qualitative research (11).

### 2.1 Research team and reflexivity

Semi-structured interviews were conducted to gather perspectives on undergoing HIV testing as GBMSM. The interview schedule was jointly designed by the research team, including: TRP (PhD), a female research fellow in sexual health with extensive experience in sexual health and conducting qualitative interviews; JJO (FAChSHM, PhD), a male sexual health physician and researcher with a special interest in increasing access to sexual health services among underserved populations; and YZ, a female PhD candidate at Monash University. Collectively, the team members have decades of experience in this field of study, varying sexual identities and cultural backgrounds, including two people of Asian ethnicity. As a result, the researchers’ clinical and cultural experiences will have influenced the type of questions they asked and affected how they interpreted the interviews.

YZ conducted all interviews. The participants had no prior relationship with YZ and were informed that the study aimed to understand knowledge, attitudes and practices of HIV self-testing among Asian-born GBMSM due to their increasing rate of HIV.

### 2.2 Recruitment

Purposive sampling was used to recruit participants who identify as Asian-born GBMSM (Table 1). Individuals of any HIV status could be involved in the study. The participants were recruited through advertising via posters within personal networks, community organizations, universities, social media platforms, snowball sampling (where interviewees were encouraged to refer interested friends) and the Melbourne Sexual Health Centre (MSHC) from 30 January to 30 May, 2023. Those interested in participating scanned a QR code on the posters to fill out a brief survey on Qualtrics, which asked about their demographic details and gender and sexual identity to verify their eligibility. They were subsequently sent a plain-language statement via email and arranged an online interview. Informed verbal consent was obtained on the day of the interview.

**Table 1:** Sampling framework, eligibility criteria and interview schedule topics.

Eligibility criteria	Gay or bisexual or other men who have sex with men (GBMSM) Cis or transgender men Aged 18 years or older Have basic or conversational English language proficiency All HIV status Born in Asia, defined as: Bangladesh, Bhutan, Brunei, Cambodia, China, East Timor, Hong Kong, India, Indonesia, Japan, North Korea, South Korea, Laos, Macau, Malaysia, Maldives, Mongolia, Myanmar, Nepal, Pakistan, Philippines, Singapore, Sri Lanka, Taiwan, Thailand or Vietnam
Sampling framework	Range of ages Length of time in Australia
Interview schedule topics	HIV and STI knowledge Preferred HIV prevention strategies HIV testing experience as a GBMSM in the country of origin HIV testing experience as a GBMSM in Australia Knowledge and experience of HIVST kit Secondary distribution of HIVST kit Partner testing using an HIVST kit

HIVST, HIV self-testing; GBMSM, gay, bisexual, and other men who have sex with men.

### 2.3 Data collection

The interviews were conducted and recorded through Zoom by YZ in English from February to May 2023 and were digitally recorded. Participants received a Zoom link to YZ’s password-protected personal meeting room, with each participant receiving a randomly generated password. Upon joining, YZ locked the meeting room to prevent others from joining. Participants had the option to enable or disable their camera during the interview. Verbal consent and the interview content were separately video recorded to maintain confidentiality. Immediately following the interviews, YZ documented contextual information regarding the interview and the participant, including non-verbal cues (e.g., hesitation, eye contact) and other pertinent details. During the interview, YZ showed and explained to the participants an information sheet on HIV self-testing. The participants were remunerated with an AUD$50 (US$33) voucher upon the completion of the interview. The audio recordings from the interviews, lacking identifying data, were sent to a professional transcription service and transcribed verbatim. Subsequently, YZ reviewed the transcripts for accuracy.

### 2.4 Data analysis

Data analysis adhered to a descriptive qualitative approach, generating data that delineate the details of the who, what, and where aspects of events or experiences (12, 13). The coding and analysis performed in this study were primarily guided by the interview-schedule questions and topics, which were informed by the relevant literature in the field and the specific clinical questions of interest to the research team. YZ undertook the initial reading and coding of each transcript. The transcripts were then imported into NVivo 12 (QSR International Pty Ltd., United States) for data management. YZ categorized the codes and constructed them into preliminary themes through an iterative coding process. A subset of transcripts was read and coded by TRP. YZ and TRP met regularly to examine the interview transcripts and discuss the codes, reaching a consensus on whether to merge some codes, remove others or generate new ones. Each transcript was re-examined, and the themes were further revised, refined, and compared to determine their similarities and differences. Periodic meetings were held to review the data and generate themes and sub-topics until 25 interviews were completed. No significant disparities in interpretation were observed. At that point, a collaborative meeting was held among the three researchers (YZ, TRP and JO) to discuss and reach a final consensus on the themes and that no more new themes were constructed (14, 15). This collaborative approach was used to develop a nuanced interpretation of the data and reduce bias in the data interpretation (16).

A descriptive analysis of the demographic data was conducted using Stata 17 (StataCorp LP, College Station, USA).

### 2.5 Data trustworthiness

To ensure the trustworthiness of the data, four criteria, namely credibility, dependability, transferability, and confirmability, were employed (17). Credibility was demonstrated through the use of well-established qualitative research methods. Prior to data collection, the interviewers established a working rapport with participants through previous interactions during the survey on Qualtrics and email correspondences. During data collection, an iterative approach was used to rephase previously asked questions. This was done to verify and gain deeper understanding of the information provided by participants based on their original responses (18). Member checking was conducted in the interview through paraphrasing and summarizing participant statements at intervals and at the conclusion of each discussion topic. The participants were provided with a copy of the study transcript for checking within a week of completing their interview to minimize recall bias. 18 participants responded with no changes, except one who replied with minor changes to the transcript. The remaining six did not provide any feedback. To ensure dependability, detailed descriptions of the study processes have been provided to facilitate replication by future researchers (18). For transferability, comprehensive details about the study methods have been included to aid in understanding and comparing the findings with those of similar studies. Confirmability was addressed by supporting the findings with verbatim quotes from study participants (18).

## 3 Results

Forty-six people expressed interest in participating in the study, of which 43 satisfied the eligibility requirements. Among eligible participants, 30 scheduled an interview; however, five did not attend their scheduled interview and could not be reached to reschedule. In total, 25 completed interviews before data collection was discontinued. No person living with HIV participated in the study. All except one participant kept their camera on during the interview. The duration of the interviews ranged from 36 to 94 min, with a median of 52 min. The age of the participants ranged from 19 to 44 years, with a median of 30 years [interquartile range (IQR): 27–34 (Table 2)]. There were 20 (80%) self-identified gay men and five bisexual men. Most were from Mainland China (*n* = 16; 64%). Eight participants (32%) reported ever having used an HIVST kit in their country of origin or Australia. Although transgender men were eligible, none participated.

**Table 2:** Participant demographics (*N* = 25).

	n (%)
**Age (years)**	
18–24	2 (8)
25–34	18 (72)
> = 35	5 (20)
**Sexual identity**	
Gay	20 (80)
Bisexual	5 (20)
**Country of origin and ethnicity**	
Mainland China	
Han	14 (56)
Manchu	1 (4)
Tujia	1 (4)
Taiwan	
Chinese	1 (4)
Singapore	
Chinese	2 (8)
Malaysia	
Chinese	1 (4)
Philippines	1 (4)
South Korea	1 (4)
India	
Hindi	1 (4)
Rajasthani	1 (4)
Vietnam	1 (4)
**Occupation**	
Undergraduate student	3 (12)
Postgraduate student	3 (12)
Working	19 (76)
**Length of time in Australia**	
<=12 months	3 (12)
1–5 years	9 (36)
6–10 years	5 (20)
>10 years	6 (24)
**Ever used HIVST**	
No	17 (68)
Yes	8 (32)
**Currently on PrEP**	
No	12 (48)
Yes	13 (52)
**Residential location in Australia by State**	
Victoria	15 (60)
New South Wales	2 (8)
Queensland	1 (4)
Western Australia	4 (16)
South Australia	1 (4)
Australian Capital Territory	1 (4)
Northern Territory	1 (4)
**Education level**	
Diploma	1 (4)
Bachelor	10 (40)
Master/Post-graduate diploma	13 (52)
PhD	1 (4)
**Initial purpose in Australia**	
Study	21 (84)
Travel	1 (4)
Work	2 (8)
Relocation with family	1 (4)
**Current visa type**	
Student	6 (24)
Work/Temporary graduate visa	6 (24)
Partner/spousal	3 (12)
Permanent residency	5 (20)
Citizen	5 (20)
**Medicare^1^**	
Yes	14 (56)
No	11 (44)
**Usual HIV prevention method**	
PrEP only	10 (40)
Condom only	9 (36)
Both PrEP and condom	3 (12)
None but sexually active	3 (12)
**Usual HIV testing venue**	
General practice	9 (36)
University clinic	1 (4)
Sexual health centre	13 (52)
Community-based testing centre	1 (4)
None	1 (4)

^1^Australia’s universal healthcare insurance scheme for residents.

HIV, human immunodeficiency virus; HIVST, HIV self-testing; PhD, Doctor of Philosophy; PrEP, Pre-exposure prophylaxis.

**Figure 1:**
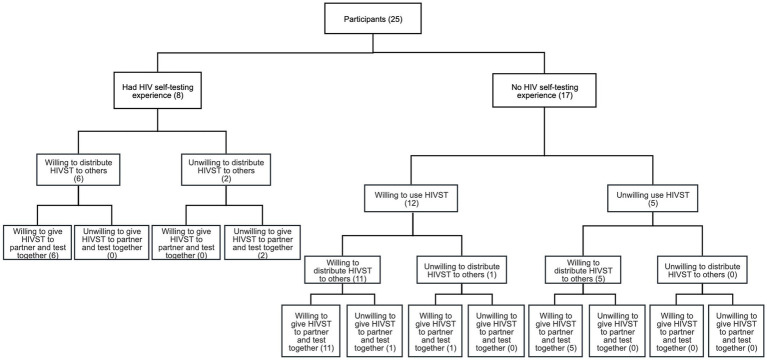
Summary of interview participants’ responses toward HIV self-testing.

The qualitative data were organized into three major descriptive themes, and eight subthemes were constructed from the study (Figure 1 and Table 3).

**Table 3:** Major themes and subthemes of knowledge, attitudes and practices of HIVST among 25 Asian-born gay, bisexual and other men who have sex with men.

Knowledge	Knowledge of HIV and HIV prevention/testing in country of origin Knowledge of HIV and HIV prevention/testing after arrival in Australia Knowledge and use of HIVST in country of origin Participants who knew about HIVST were exclusively from China Point of sex testing, testing after high-risk exposure Avoid facility-based testing due to perceived stigma Knowledge and use of HIVST in Australia Little to no awareness of the availability of HIVST in Australia Avoid facility-based testing due to internalized stigma, migration or confidentiality concerns
Attitude	Willingness to use HIVST among non-HIVST users Yes: Quick, discreet, prompt results “Prefer to process HIV diagnosis by themselves, not in a clinical environment” No: Preference for conventional serology Prefer to undergo STI testing at the same time On daily PrEP, thus HIV testing is already done every three months HIVST is pricey Low perceived self-risk of HIV Accessibility of HIVST in Australia Preference for anonymous access to HIVST kits
Practices	Opportunities to use HIVST/ situations that they would find HIVST useful Traveling (including going back to the country of origin) Point-of-sex testing Interim testing when taking on-demand PrEP Willingness toward secondary distribution Yes: Distribute to gay friends and partners No: Fear of being judged and the sensitive nature of HIVST Willingness toward partner testing Yes: Point-of-sex testing with casual sex partners (i.e., one-night stand) for self-protection, for peace of mind With a regular partner (i.e., fuckbuddy, boyfriend, husband, partner) if engaging in condomless sex Within an open relationship—builds trust As a baseline test within a monogamous relationship No: Might not be feasible to persuade a casual sex partner to test Kill the mood Opportunities/ recommendations to promote HIVST as told by HIVST users and non-users Avenues to promote HIVST Information needed from HIVST Where, how, accuracy Translated into different languages, pictorial, QR code

HIV, human immunodeficiency virus; HIVST, HIV self-testing; PhD, Doctor of Philosophy; PrEP, Pre-exposure prophylaxis.

### 3.1 Knowledge

#### 3.1.1 Knowledge of HIV and HIV prevention/testing in their country of origin

All the participants reported limited sexual health knowledge (i.e., HIV testing window period, HIV prevention such as PrEP, post-exposure prophylaxis) before they arrived in Australia. Most reported that discussions on sexual health and HIV were rare or discouraged in their respective countries of origin. HIV was perceived as a ‘dirty’ disease, and HIV-related stigma remained widespread within their communities in their country of origin. They believed that getting HIV was akin to a death sentence and there was no treatment upon diagnosis. Although some participants were aware of the importance of HIV testing, they were unsure where to get tested.

*People are very conservative. We are ashamed to talk about it [HIV]. It's considered a taboo. I had many questions. I had never spoken to a doctor or a healthcare professional before about all these. Back then, I didn’t know about PrEP… The schools and universities, they don't provide any sexual education to students in India. –Participant 25, 19 years old, India.*

*They [the people in the country of origin] make this word [HIV] really serious. They keep telling stories of people who die from this disease and there is no medicine to treat it. Once you get it, you die from it. –Participant 02, 25 years old, China.*

#### 3.1.2 Knowledge of HIV and HIV prevention/testing after arrival in Australia

Nearly all participants reported increased sexual health knowledge after they arrived in Australia, particularly among those who regularly visited sexual health clinics for HIV testing. Some participants learnt about PrEP as a HIV prevention method after arrival in Australia. They appreciated the sexual health staff’s patience and expertise in addressing their concerns and educating them on HIV prevention strategies. Most were introduced to sexual health clinics by their friends or partners.

*Every time I go there [MSHC], I ask one question and I get new information. Along the way, then I start to learn how HIV is transmit between people and then start to learn some words, Like U=U. Or have some PrEP before high-risk sex and how HIV is related to high-risk sex. My fear about HIV got less and less and less. I know I need to get tested every 3 months or something like that. –Participant 02, 25 years old, China.*

#### 3.1.3 Knowledge and use of HIVST in country of origin

Eight participants from China had previously utilized HIVST kits in their country of origin. Each participant reported a precipitating factor that motivated them to undergo an HIV self-test. Most indicated that they used HIVST for point-of-sex testing before engaging in sex or following a potentially high risk of exposure. Some opted for HIVST to circumvent facility-based testing, citing concerns about confidentiality, privacy and the stigmatizing attitudes of healthcare personnel at testing centers.

*I bring the self-testing kit to hook up. So before we have some intimacy, we just do the test first. Especially when I meet some new people, definitely I have to get tested. –Participant 14, 27 years old, China.*

#### 3.1.4 Knowledge and use of HIVST in Australia

Seventeen participants were unaware of HIVST before their interviews, and most, upon learning about HIVST during the interviews, were interested in learning more about it. Only two were aware of the availability of such kits for purchase in Australia, and both were prior users of HIVST in their country of origin.

*I have never heard of it (HIV self-testing kit) until I read that document (HIV self-test information sheet) that you sent me. I don’t know I can buy that in Australia. Before this. I don’t know where they have that because I’ve never seen it before at the pharmacy. –Participant 15, 29 years old, China.*

Furthermore, one participant who practices as a general practitioner (GP) in Australia was unaware that HIVST was available in Australia. He felt that more work was needed to increase awareness of HIVST among primary-care physicians in Australia.

*Until today I didn't know it was an option for self-testing. I'm not sure ASHM [Australasian Society for HIV, Viral Hepatitis and Sexual Health Medicine] had even told practitioners that this is an option. More work needs to be done around raising awareness. –Participant 05, 36 years old, Singapore.*

Several participants who had previously utilized HIVST chose to continue using these kits after arriving in Australia, having brought them from their country of origin. This was motivated by concerns surrounding disclosing their medical information to authorities. They identified medical confidentiality, visa and immigration aspirations as complicating factors that deterred them from accessing facility-based testing. The fear of potentially discriminatory immigration practices and being deported from Australia because of their HIV status, if they tested positive, contributed to choosing to self-test.

*I was still a student at the time and I'm like, what if I catch something and then my visa will be cancelled and everything? What if they (healthcare staff) report me? –Participant 11, 29 years old, China.*

One participant reported continuing to use HIVST, although he visits a GP for other health issues.

*I see my GP for my eczema … One time I tried to ask about HIV testing and PrEP, she asked me if I was having sex with boys. I felt bad. I didn’t want to tell [her] my sexual identity so I said no. They don't really say something weird, but I can feel from their eyes and their postures. So I just test myself. –Participant 22, 36 years old, China.*

### 3.2 Attitudes toward HIVST in Australia

#### 3.2.1 Willingness to use HIVST among non-HIVST users

More than half of the participants who had not used HIVST before were open to HIVST, with a particular inclination being observed among individuals not on pre-exposure prophylaxis (PrEP). They agreed they would use HIVST had they been aware of it. The most cited motivation to use HIVST was its convenience, discretion, and prompt results. HIVST was seen as eliminating the prolonged experience of dread and ‘anxiety [associated] with waiting for conventional serology results’.

*Yes, I would love to try it [HIVST]. It's quick, just 15 minutes. Otherwise, I have to wait for one week for the results [conventional serology]. And during that one week, even I knew that I wouldn't get HIV, but I still worry. –Participant 20, 27 years old, China.*

One participant had purchased an HIVST kit in Australia after seeing an advertisement for it on a dating app (Grindr), as he preferred to handle his HIV diagnosis alone rather than in a clinical setting because the hospital environment is stressful for him.

#### 3.2.2 Unwillingness to use HIVST among non-HIVST users

Most participants already engaged in sexual health care expressed reluctance toward utilizing HIVST and were inclined toward conventional serology. This was either due to their adherence to regular testing because of taking daily PrEP or their desire to undergo testing for other sexually transmitted infections (STIs) besides HIV.

*I test HIV as well as other STIs in every three months at least. It's mandatory to get my PrEP prescription here. That took away the need to do self-testing after sex. –Participant 01, 38 years old, Taiwan.*

Cost was a critical factor in their decision-making process to purchase an HIVST kit. They were reluctant to pay for an HIVST kit, citing the availability of cost-free conventional testing as a viable alternative for those attending sexual health clinics. Nevertheless, upon being queried about their willingness to use HIVST if it could be provided free of charge, all indicated a readiness to do so.

*Because pathology test is covered by Medicare now, as a PR. So I do not have to pay anything, whereas you have to pay $30 for a self-testing kit. But if it’s free, why not? I would love to try. –Participant 02, 25 years old, China.*

Other concerns cited include the accuracy of the HIVST and the long window period compared to conventional serology (after knowing about it at the interview).

*I wouldn't, because accuracy is not that high or like a hundred percent. Also because of the window period. I want to get an accurate result. I can't just stop having sex for 3 months. –Participant 09, 33 years old, China.*

A few had concerns and a lack of confidence in performing the HIVST correctly.

*I would let a professional do it. I don't trust myself cutting myself open. –Participant 07, 31 years old, China.*

Two participants perceived their risk of contracting HIV as low, as they were in monogamous relationships, and hence did not see the need for HIV testing. One participant was averse to HIVST and refrained from undergoing any HIV screening. The primary factor leading to this was a fear of knowing his HIV status and anticipated social isolation from his friends and community if he tested positive for HIV.

*"Okay, I'm not going to go for HIV test because I'm clean. I can be friend with you, " because they'll be like, "Oh, my God, if you have HIV don't touch me." And you don't want to be treated differently. –Participant 18, 26 years old, China.*

#### 3.2.3 Accessibility of HIVST in Australia

Among participants who were interested in HIVST, there was a preference for more anonymous access. Some expressed that they would opt for HIVST; however, they hesitated due to the associated stigma and apprehension regarding potential judgment if they were observed purchasing the test at a pharmacy. For one participant, the hesitancy to purchase HIVST was related to internalized stigma associated with HIV and HIV testing:

*There is some stigma. It could happen that when I'm speaking [to the pharmacist], some of my friends could be there or could hear what I'm asking for. Obviously, even if you have left your country, still, that thing [stigma associated with HIV and HIV testing] carries with you. It's not an on/off switch where I can just turn it off. –Participant 16, 28 years old, India.*

Nevertheless, nearly all indicated that if HIVST kits were available through retail, they should be available off the shelf rather than behind the counter. Placing HIVST kits on the shelves would enhance their visibility, thereby heightening public awareness of the availability of HIVST. Additionally, they feared embarrassment or stigma if they approached the pharmacist.

*Why make it another step harder for people that doesn't want to get find out. Why not just put it [HIVST kit] on the shelf? –Participant 08, 34 years old, Malaysia.*

A few participants suggested using vending machines for purchasing HIVST kits.

*You know those coin machine selling condoms, can do the same for HIVST. Put in universities. I can buy it when there's no people and put it in my wallet or my pocket to hide, very quickly. –Participant 04, 27 years old, China*.

### 3.3 Practices

#### 3.3.1 Opportunities to use HIVST

Participants who indicated a desire to use an HIVST kit expressed a willingness to do so under certain circumstances, such as when traveling or when access to facility-based HIV testing is limited. Some pointed out they may opt for HIVST when returning to their country of origin to circumvent facility-based testing.

*Because firstly, during travelling, because it's easy and convenient. If I'm a tourist, I can't just stay for another week to wait for the results. Secondly, if I'm in India, then I would use a self-test, do it in a locked room with nobody else there. So I don’t have to go to the clinic. –Participant 25, 19 years old, India.*

Some spoke about considering HIVST for point-of-sex testing or situations in which they engaged in sexual activity that increased the risk of HIV transmission.

*You need to keep one or two around you just in case there is always a moment that you or your partner take off the condom. Especially if you go to an orgy and people have something [an STI/HIV] and just protect yourself. –Participant 06, 25 years old, China.*

A few said they would use HIVST as interim testing when on PrEP, especially if they used on-demand PrEP and perceived conventional serology every 3 months as inconvenient.

*If you're taking on demand, you can probably stretch it out to six months or even longer. It would be nice to have the option to do the HIV self-test without having to go to the doctor. –Participant 05, 36 years old, Singapore.*

#### 3.3.2 Willingness to engage in secondary distribution

When the possibility of peer distribution of HIVST kits was raised, most demonstrated a positive attitude toward distributing HIVST to their gay friends or sexual partners. Some suggested they would most likely distribute the HIVST kits to their friends and sexual partners who are not using PrEP or who they know are engaging in sexual activities where HIV transmission is likely to occur. The participants considering distributing the HIVST kits tended to be socially connected to the gay community and not in a regular partnership.

*Yeah, but if I can prioritize, I would distribute them to someone who's sexually active and who has more than one partner and that I know that are not taking PrEP. –Participant 03, 26 years old, South Korea.*

Some were optimistic but also cautious about distributing HIVST kits, citing fear of being judged and the sensitivity of HIVST. A participant said that providing HIVST could be seen as being judgmental toward someone’s sexual behavior.

*I'm kind of implying, "You are at risk of having HIV, so that's why I'm giving you this testing kit." I don't want my friend to think that I'm judging them. –Participant 24, 32 years old, Vietnam.*

#### 3.3.3 Willingness toward partner testing

With a few exceptions, most were willing to use an HIVST kit if requested by a sexual partner. More than half would consider asking their sexual partner to use an HIVST kit, particularly if they are not on PrEP. Among those who opt for partner testing, most would conduct HIVST in the company of their casual sexual partners (e.g., one-night stand, sex with random strangers). They perceived that using the HIVST kits together would give them a sense of psychological well-being and enhance their sexual experience by confirming their partner’s HIV-negative status.

*Everyone can claim, "Yeah, yeah, I am, so on. I am (HIV) negative. For safety purposes, it [partner testing] would be really nice. I think it's a matter of making it as a practice, especially if it's a casual sex. –Participant 10, 34 years old, Philippines.*

However, there were also concerns regarding the feasibility of persuading their sex partner to engage in HIVST. A few were worried whether conducting the test before sex would ‘kill the mood.’

*It may kill the vibe a little bit because when people are just going there for sex and if you don't let them know beforehand that you wanted to test together, then it might change the dynamics. –Participant 01, 38 years old, Taiwan.*

Some who were in a relationship and/or have regular sex partners (i.e., someone they engage in sexual activities regularly) would consider HIVST. These participants perceived that using HIVST together would demonstrate trust and a sense of security in their relationships. Among these participants, those in open relationships expressed the benefit of HIVST. One participant, who was in a monogamous relationship, saw HIVST as a mechanism to solidify his relationship.

*I will feel very safe, and I will love him more [if we use HIVST together]. It will help to build trust. If we use it regularly, there's one more level of trust… It builds your relationship. –Participant 04, 27 years old, China.*

One participant was very supportive toward partner testing as he recounted his experience of doing partner testing with an HIVST kit in his country of origin, wherein he discovered that the other individual had a reactive test result.

*In my last relationship, we were doing the partner testing [with HIVST kits]. So he was positive, and I was negative at that time. I freaked out. We always had unprotected sex. I was scared to death. –Participant 14, 27 years old, China.*

#### 3.3.4 Opportunities/recommendations to promote HIVST as told by HIVST users and non-users

A universal theme was that a more proactive approach to publicizing HIVST would be beneficial in increasing the awareness and uptake of HIVST. Some highlighted the need for non-judgmental HIVST advertising that includes all people, regardless of sexual orientation.

*But in a way that it will not make people think, "Oh, it's just only for the gay people." I think it [HIVST] should be promoted just to general public, not just to gay community. More non-judgmental would be really cool. –Participant 08, 34 years old, Malaysia.*

Participants expressed a desire for additional information regarding the availability of HIVST kits in Australia, instructions on their usage, and the accuracy of the test. Some hoped the instructions would be translated into multiple languages and with visual aids.

*Different languages will make it easier, not everyone is fluent in English, and they might not understand the professional terms. It's very important to put different languages. And it feels more personal, as well. –Participant 02, 25 years old, China.*

## 4 Discussion

This study makes a unique contribution to the literature by canvassing the views of participants from various Asian backgrounds on HIVST and drawing on past testing experiences to shed light on their knowledge and attitudes toward HIVST. Our analysis revealed that all participants had limited sexual health knowledge before they arrived in Australia, and most were unaware of the availability of HIVST in Australia before the interview. Upon learning more about HIVST, many subsequently expressed a willingness to use HIVST. However, they mostly would not alter their current HIV testing routines (preferring conventional serology for its free cost, accuracy, and concurrent STI testing) but could envision using the kits in specific situations, including during casual encounters, while traveling, or for interim testing when taking on-demand PrEP. Almost all were open to distributing HIVST to their gay friends or sexual partners, especially those they knew were not engaged in sexual health care. They were also interested in partner testing, primarily with non-regular sexual partners for peace of mind or with regular sexual partners when engaging in condomless sex.

Among those with previous experience with HIVST, most utilized them for point-of-sex testing or to avoid facility-based testing due to confidentiality and privacy reasons or a fear of judgment on the part of healthcare staff. One of the main facilitators of HIVST was HIV-related stigma, which was experienced at the individual and community levels. The interviews revealed an interplay between HIV-related stigma and attitudes toward HIVST. We found that GBMSM were dissuaded from undergoing conventional HIV testing due to prior experiences of stigma in their countries of origin. Much of this stigma can be attributed to misconceptions associated with HIV in countries of origin with conservative cultural systems. For many, there was a fear of the judgments of others, particularly health service providers, if they were to test. The confidentiality of undergoing conventional serology and disclosing sexual practices were subjects of concern. The possibility of such information being communicated to families, community members, or immigration authorities through health services was also raised. In particular, within the migration context, newly arrived individuals who intend to pursue permanent residency in the future tend to avoid seeking sexual healthcare services at facilities (5) and, instead, opt for HIVST.

One key highlight in the interviews was that among prior users of HIVST, most were cognizant of the concept of a window period in HIV testing, except for one or two participants who had heard of the term but were unaware that HIVST has a window period of 3 months. Hence, they reported using HIVST for point-of-sex testing or testing immediately after a high-risk exposure, confirming their misconception. Recognizing the possibility of a false negative result when using HIVST soon after a possible exposure due to the window period is important. Furthermore, given the poor awareness of undetectable = untransmissible (19), engaging in point-of-sex testing could result in partner rejection for an individual with an undetectable HIV viral load.

Our study found that convenience, discreetness and reduced wait time for the results were cited as the main facilitators of HIVST. Previous work has trialed self-testing kits in Australia, with encouraging levels of willingness to use and good uptake (20–22). In an online blood-based HIVST dissemination project led by an Australian community peer HIV organization, it was found that most (79.4%; 726/914) cited convenience as their reason for using HIVST, with a further 29.3% (268/914) of orders being due to consumers not having time to go to the clinic for a test (21), showing that MSM were receptive to HIVST for the reasons cited above. Our study also revealed that among participants who were accepting of HIVST, most preferred anonymous access to HIVST and were averse to purchasing HIVST kits over the counter in pharmacies. A discrete choice analysis of HIVST among men who have sex with men in Australia reported a preference for online distribution or kits available off the shelf (23). Hence, it is pertinent to explore other flexible models of HIVST delivery in Australia that provide greater convenience and best suit the needs of Asian-born GBMSM. A body of research has highlighted that the secondary distribution of HIVST through social networks may increase HIV diagnoses and help identify additional people with HIV (24). Furthermore, the World Health Organization recommends that social network–based testing approaches (SNA) be offered as an additional approach to HIV testing for key populations (25). This approach is deemed acceptable, feasible and resource efficient, particularly when targeted at individuals with high ongoing HIV risk (26). Our findings confirmed that almost all participants were open to distributing HIVST to their gay friends or intimate partners, especially those engaging in sexual practices where HIV is likely to be transmitted and people who did not engage in sexual healthcare. Further research is needed to pilot the use of SNA for the secondary distribution of HIVST to increase uptake among Asian-born GBMSM.

Importantly, our study revealed that a lack of awareness of HIVST was a major barrier to the uptake of HIVST in Australia. Upon learning about HIVST during the interviews, many participants were willing to use it, highlighting the importance of developing interventions to support HIVST awareness and distribution. More efforts are needed to increase knowledge of HIVST in Australia, such as social media campaigns or utilizing pamphlets and posters framed with positive sexual-health messaging and disseminated through schools, universities and communities. A study of HIVST multimedia campaigns among young people reported a 32% increase in the awareness of HIVST and a 19% increase in the uptake of HIVST in South Africa, showing that HIV campaigns have a positive effect on the awareness and uptake of HIVST (27). Furthermore, given the unique role of culture and social influences on individual perceptions of risk and additional concerns about discrimination, there is a need for community-specific approaches to address these factors (28). A previous qualitative study of 24 Asian-born GBMSM reported that they face racial discrimination but being engaged with the gay community can increase sexual health knowledge and encourage better sexual health practices (6). Therefore, it is imperative that all communities are adequately represented in the HIV response without perpetuating further differentiation or attributing fault to particular ethnic groups. In line with good health-promotion practice, consultation and engagement with the community are necessary to ensure that the community has buy-in regarding forthcoming projects and an opportunity to speak about the issues that most affect them (29).

Some study participants who were interested in HIVST but had some concerns cited cost as a deterrent to acquiring an HIVST kit. Consistent with previous Australian (22, 30) and international research (31), the sale of HIVST kits is likely unable to meet the needs of those who want self-testing. According to Bell et al., the median amount that participants were willing to pay was AUD$20 (21). This is also consistent with results from an Australian discrete choice experiment which reported a preference for HIVST kit costing less than AUD$40 (23). This suggests that the existing commercial market-driven HIVST distribution model may be a barrier to uptake and that, to be effective, HIVST programs will require external funding or subsidization to enhance population coverage and access.

In addition, there was a perception that general practitioners (GPs) needed to take a more proactive role to encourage the uptake of HIVST, including providing information about HIVST and addressing concerns of confidentiality and treatment availability. Indeed, GPs play a vital role in ensuring the early diagnosis of HIV and a transition into care, particularly among communities that may not have access to sexual health clinics (32). Challenges exist in convincing GPs to encourage testing uptake, including limited knowledge of HIV and a reliance on HIV-specialist GPs to educate clients on HIV (33). Furthermore, no online training courses on HIVST are currently available for GPs in Australia. Targeting GPs working predominantly with vulnerable communities regarding HIV testing, including messages of prevention and accurate messaging about the window period for point-of-sex testing, should be considered by medical bodies and state governments. Opportunities outside of general practices should be explored by non-government organizations, including considering whether locations such as community centers, sporting events, and cultural events may be effective locations for testing (34).

The main limitation of our study is that we could not recruit men to represent all Asian countries; our sample was predominantly from China and relatively few from other countries. However, like all qualitative data, the qualitative component was not meant to be generalizable to the wider population of overseas-born GBMSM. Rather, our study added depth and understanding to the overall body of knowledge on HIVST by providing insights into HIVST from a specific group, namely Asian-born GBMSM.

## 5 Conclusion

Our study highlights the complexity and multifaceted nature of barriers to and facilitators of HIVST in Asian-born GBMSM living in Australia. Although HIVST represents an opportunity to engage with people outside of conventional healthcare settings, interventions intended to increase the uptake of HIVST among Asian-born GBMSM in Australia must be multi-strategic and aimed at the individual, community and policy levels. In addition to addressing the intersecting inequities of sexual identity, key policy mechanisms to address HIV-related stigma must be co-designed and implemented with input from key populations. This includes culturally appropriate education and information; the involvement of Asian-born GBMSM community members and addressing policy and legislative barriers to make HIVST easier to access for all. The continued implementation of multi-level interventions to facilitate early and routine HIV testing is of utmost importance in this area of high HIV prevalence and late diagnosis, especially in an era of universal testing and treatment. Using novel diagnostics (i.e., HIVST) can be an additional testing approach to increasing HIV testing for key populations.

## Data availability statement

The original contributions presented in the study are included in the article/supplementary material, further inquiries can be directed to the corresponding author.

## Ethics statement

The studies involving humans were approved by Alfred Hospital Ethics Committee, Australia (Project no: 684/22). The studies were conducted in accordance with the local legislation and institutional requirements. The participants provided their written informed consent to participate in this study.

## Author contributions

YZ: Conceptualization, Formal analysis, Investigation, Methodology, Software, Writing – original draft, Writing – review & editing. EC: Writing – review & editing. BS: Writing – review & editing. DW: Writing – review & editing. MS: Writing – review & editing. NM: Writing – review & editing. DO'D: Writing – review & editing. PK: Writing – review & editing. JO: Conceptualization, Supervision, Writing – review & editing. TP: Conceptualization, Supervision, Writing – review & editing.
